# Visual Responsivity in Autism: Measuring Visual Responses in Autistic and Non‐Autistic Adults Using Psychophysics, fMRI, and EEG

**DOI:** 10.1002/aur.70335

**Published:** 2026-07-31

**Authors:** Daniela L. Seczon, Kristin M. Woodard, Tamar Kolodny, Hannah M. Rea, Mark Pettet, Sara Jane Webb, Scott O. Murray

**Affiliations:** ^1^ Department of Psychology University of Washington Seattle Washington USA; ^2^ Department of Psychology Ben‐Gurion University of the Negev Beersheba Israel; ^3^ Department of Psychiatry and Behavioral Sciences University of Washington Seattle Washington USA; ^4^ Seattle Children's Research Institute, Center for Child Health, Behavior and Development Seattle Washington USA

## Abstract

Autistic individuals frequently report heightened sensitivity to visual stimuli, often described as discomfort in environments with bright or flickering lights. These experiences are hypothesized to reflect underlying differences in sensory gain or neural hyperexcitability; however, findings across studies have yielded mixed results, likely due to methodological variability. This study aimed to evaluate whether group differences in contrast‐dependent neural and behavioral responses to a visual stimulus would be observed across complementary methods: psychophysics, fMRI, and EEG. Thirty‐one autistic and twenty‐seven non‐autistic adults completed experimental sessions in which they passively viewed bilaterally presented 6 Hz counterphase flickering checkerboards at high (100%) and low (2%) contrast. Neural responses were measured using the blood oxygenation level‐dependent (BOLD) signal from fMRI and steady‐state visual evoked potential (SSVEP) from EEG. Contrast robustly modulated responses across experiments, eliciting higher neural responses; however, no group differences emerged in BOLD or behavioral thresholds. The only significant group difference was observed in SSVEP amplitudes, with autistic individuals showing significantly higher neural entrainment to the flickering stimulus than their non‐autistic counterparts. Moreover, SSVEP amplitudes were associated with BOLD responses to the same low contrast visual stimulus, suggesting convergence between frequency‐locked EEG responses and hemodynamic activity. SSVEP amplitudes were additionally associated with self‐reported measures of hypersensitivity, linking heightened neural entrainment to individual differences in sensory experiences. They also showed a differential relationship with perceptual thresholds across groups. These findings show that differences between autistic and non‐autistic participants were more evident in frequency‐locked neural responses to periodic visual input, as measured by SSVEPs, and that these responses were meaningfully related to visual cortical BOLD activity and sensory hypersensitivity.

## 1 Introduction

Autism spectrum disorder (ASD) is a neurodevelopmental condition characterized by differences in social communication and the presence of restricted and repetitive behaviors (American Psychiatric Association 2013). Sensory processing differences are among the core features of autism, which can include both hypo‐ and hypersensitivity (Ben‐Sasson et al. 2009; Brown et al. 2001; Rogers and Ozonoff 2005). These sensory differences are highly prevalent (Ben‐Sasson et al. 2009; Brown et al. 2001; Crane et al. 2009; Leekam et al. 2007; Rogers and Ozonoff 2005; Tomchek and Dunn 2007) and are known to impact an individual's well‐being across the lifespan (Dellapiazza et al. 2018; Hilton et al. 2010; Neufeld et al. 2021). However, despite their prevalence and relevance, their underlying neurobiological mechanisms remain poorly understood.

Sensory differences in autism span across modalities, with the visual domain being one of the most frequently reported (Brown et al. 2001; Leekam et al. 2007; Nagib and Williams 2017). Autistic individuals report heightened visual sensitivity, such as experiencing discomfort in environments that feature bright or flickering lights (Brown et al. 2001; Chamak et al. 2008; Jones et al. 2003; Leekam et al. 2007; Nagib and Williams 2017; Robertson and Simmons 2015). Autistic adults have described the debilitating consequences of sensory differences and emphasize the need for greater scientific attention and targeted interventions to address these challenges (Cage et al. 2024). These clinical observations motivate the use of experimentally controlled paradigms that assess responsivity in early stages of visual processing, including responses to systematic changes in visual input strength.

In the present study, visual input strength was manipulated by varying stimulus contrast. Contrast refers to the luminance difference between light and dark regions of a stimulus, and neural and behavioral responses in early visual processing are strongly influenced by contrast. The systematic increase in visual responses with increasing stimulus contrast provides a useful tool for examining how strongly the visual system responds to changes in input strength. Behaviorally, contrast sensitivity refers to the ability to detect small differences in luminance contrast, with lower detection thresholds indicating greater sensitivity (Campbell and Robson 1968). At the neural level, measuring responses across contrast levels provides a way to examine contrast‐dependent response scaling, a process closely related to contrast gain control, or changes in neural response magnitude as a function of stimulus contrast (Boynton et al. 1999; Ohzawa et al. 1985). Therefore, in the present study, visual responsivity is defined as the magnitude and contrast‐dependent scaling of stimulus‐evoked neural and behavioral responses.

Flickering visual stimuli are well‐suited for studying visual responsivity because they provide temporally regular input that elicits reliable responses in the visual system (Herrmann 2001; Norcia et al. 2015). They are also clinically relevant because autistic individuals often report discomfort in visually intense environments, including environments with bright or flickering visual input (Parmar et al. 2021). Therefore, flickering stimuli provide an experimentally controlled approach for testing visual response properties that may be relevant to everyday sensory experiences.

The same periodic stimuli also provide a useful bridge across EEG and fMRI measures. With EEG, responses to periodic stimulation can be quantified using steady‐state visual evoked potentials (SSVEPs), defined here as EEG signal amplitude at the flicker frequency. SSVEPs provide a measure of stimulus‐locked synchronization, or entrainment to periodic input, defined here as the extent to which neural activity follows the temporal structure of the visual stimulus (Herrmann 2001; Norcia et al. 2015). Measuring SSVEP amplitude across contrast levels therefore provides a way to examine how temporally precise visual responses scale with input strength. The same flickering black‐and‐white checkerboard stimuli can also be used with fMRI, where they robustly elicit BOLD responses in early visual cortex and are commonly used in visual localizer and retinotopic mapping paradigms (Boynton et al. 1996; Engel 1997; Sereno et al. 1995). In contrast to EEG, fMRI BOLD responses reflect slower, spatially localized hemodynamic consequences of neural activity accumulated over seconds, capturing overall response magnitude rather than temporal precision or phase locking. Thus, the same flickering stimulus across EEG and fMRI allows for complementary measurements of visual responsivity: EEG captures frequency‐specific temporal tracking of the stimulus, whereas fMRI captures spatially localized response magnitude in early visual cortex.

A complementary behavioral approach is to measure how sustained visual stimulation changes subsequent visual sensitivity. Visual adaptation refers to the reduction of visual responses following sustained exposure to a stimulus. This mechanism is thought to help maintain sensitivity to environmental changes by reducing responses to repeated or constant input (e.g., Kohn 2007). Psychophysical adaptation paradigms leverage this process by measuring perception after exposure to an adapting stimulus. In the context of contrast detection, stronger adaptation is expected to make subsequent detection of similar stimuli more difficult, resulting in higher contrast detection thresholds (e.g., Gardner et al. 2005; Greenlee and Heitger 1988). Thus, whereas EEG and fMRI measure neural responses during visual stimulation, psychophysical adaptation provides a behavioral index of how sustained visual input alters subsequent sensitivity, capturing the downstream perceptual consequences of visual adaptation.

Prior studies of early visual processing in autism have produced mixed evidence for enhanced, reduced, or comparable visual responsivity across EEG, fMRI, and behavioral measures. Several steady‐state EEG studies suggest differences in contrast‐dependent responses in autism. For example, Takarae et al. (2016) reported steeper SSVEP contrast‐response functions and elevated amplitudes at high contrast to flickering gratings in autistic participants. In contrast, Weinger et al. (2014) reported reduced responses at low contrast and increased neural noise in autistic children. Other work using fast periodic visual stimuli in autistic adults has found no significant group differences in neural detection thresholds for contrast or spatial frequency manipulations, despite elevated self‐reported sensory sensitivity (Sapey‐Triomphe et al. 2023). Related studies have reported selective differences in early visual response properties, including harmonic‐ and spatial frequency‐specific reductions in autistic children and adolescents (Pei et al. 2014), and differences in harmonic components or contrast‐response functions associated with developmental stage and autistic traits (Jackson et al. 2013; Vilidaite et al. 2018).

Findings from event‐related potential (ERP) and fMRI studies are similarly mixed. ERP studies examining early visual components have reported larger amplitudes in autistic individuals (Frey et al. 2013), reduced amplitudes (Brandwein et al. 2013; Kovarski et al. 2019), and no significant group differences (Brandwein et al. 2015; Butler et al. 2017; Milne 2011; Milne et al. 2009). Similarly, fMRI studies have reported elevated activation in occipital regions during visual tasks (Green et al. 2013; Samson et al. 2012), lower visual cortical responses (Haigh et al. 2015), or no significant group differences in responses to visual stimulation (Dinstein et al. 2012; Murray et al. 2020). Psychophysical findings are also mixed, with some studies reporting no group differences in contrast sensitivity functions across spatial frequencies (Koh et al. 2010; Milne and Buckley 2010) or in flicker contrast sensitivity (Pellicano et al. 2005), and other work reporting selective differences in responsivity to high‐spatial frequency gratings, with autistic adults showing enhanced responsivity (Kéïta et al. 2014).

One challenge in interpreting this mixed literature is that prior studies have differed in participant characteristics, stimulus properties, and measurement modality. Different methods are sensitive to distinct neural processes and timescales; for example, fMRI captures hemodynamic changes over seconds, whereas EEG resolves changes in electrical activity at the millisecond level. Likewise, variation in stimulus characteristics, such as contrast, spatial frequency, and temporal frequency, can substantially alter the neural and behavioral responses elicited, particularly for periodic flickering stimuli that engage frequency‐specific neural responses (Herrmann 2001; Norcia et al. 2015). As a result, it remains unclear whether inconsistencies across studies reflect true heterogeneity in visual responsivity in autism or methodological differences across studies. The present study addressed this issue by measuring visual responsivity with psychophysics, fMRI, and EEG in the same individuals while using the same periodic flickering checkerboard stimulus across methods. This design allowed us to compare complementary indices of visual responsivity while minimizing differences in stimulus properties across experiments.

The overarching research question of this study was whether autistic and non‐autistic individuals differ in visual responsivity to a periodic visual stimulus, and whether such differences are consistent across complementary measurement modalities. Here, visual responsivity refers specifically to the magnitude and contrast‐dependent scaling of neural and behavioral responses to a flickering checkerboard stimulus. We examined group differences in fMRI BOLD responses, SSVEP amplitudes, and adaptation‐related contrast detection thresholds. Based on prior reports of heightened visual sensitivity in autism and previous evidence for altered visual responses, we predicted that autistic individuals would show greater stimulus‐evoked neural responses than non‐autistic individuals, particularly in measures that directly capture responses to the flickering stimulus. By manipulating stimulus contrast, we tested how neural synchronization, fMRI response magnitude, and adaptation‐related changes in sensitivity scale with the strength of visual input. We also examined associations between neural responses and self‐reported sensory traits, predicting that individuals with greater visual responsivity would report greater sensory sensitivity. Finally, we explored associations across methods to investigate whether individual variability in stimulus‐evoked responsivity generalizes across neural and behavioral responses to periodic visual stimulation.

## 2 Methods

### 2.1 Participants

Thirty‐one autistic and twenty‐seven non‐autistic adults participated in a multi‐session experimental procedure involving psychophysics, fMRI, and EEG sessions. Participants completed the three experimental sessions in under 2 months on average (autistic: M = 47.4 days, SD = 43.6; non‐autistic: M = 45.4 days, SD = 39.7; *t*(55.0) = 0.18, *p* = 0.85). Sample size varied slightly across experiments due to participant dropout and data exclusion; values are presented in Table 1. Participants were recruited through flyers distributed in the Seattle metro area, social media advertisements, and online postings through the Institute of Translational Health Sciences (ITHS) Participate in Research and Biomedical Informatics services. Inclusion criteria for the autistic group included a prior ASD diagnosis and documentation, and confirmation by clinicians through a telehealth version of the ADOS‐2 Module 4 (Hus and Lord 2014; Lord et al. 2012) and meeting DSM‐IV criteria for ASD. ADOS‐2 comparison scores were calculated according to procedures outlined in Hus and Lord (2014), providing a standardized value that accounts for age and language level. Autistic adults also reported having ADHD (51.6%) and Tourette's (3.2%). Inclusion criteria for non‐autistic individuals required (1) no history of ASD or NDD diagnosis, (2) no ASD diagnosis in first‐degree relatives, and (3) no elevated autistic traits via self‐report on the Autism‐spectrum Quotient (AQ‐28; Baron‐Cohen et al. 2001; Hoekstra et al. 2011) (raw score < 70).

**TABLE 1:** Sample size per experiment.

Group	Psychophysics	fMRI	EEG	Total
Autistic	30	30	29	31
Non‐Autistic	25	27	23	27

Exclusion criteria for all participants included (1) major psychiatric concerns (e.g., schizophrenia or other psychotic disorders), (2) genotypically defined neurodevelopmental disorder, (3) a history of seizures, neurological disease or serious head injury, and (4) sensory or motor impairment that would limit the completion of the study protocol. All participants had normal or corrected‐to‐normal vision and had a full scale IQ ≥ 85 derived from the four‐subtest (Full Scale 4) Wechsler Abbreviated Scale of Intelligence Second Edition (WASI‐II, Wechsler 2011). In cases when a participant had missing data from one subtest, the two‐subtest (Full Scale 2) estimate was used instead; this adjustment was necessary for only one autistic participant. The Social Responsiveness Scale (SRS, Constantino and Gruber 2005) was used to assess autistic traits in both groups. Participant characteristics are presented in Table 2 (additional demographic characteristics can be found in Supplemental Table 1).

**TABLE 2:** Demographic characteristics of the sample.

Measure	Autistic	Non‐autistic	Group difference
	Mean ± SD, range or *N*	Mean ± SD, range or *N*	Test statistic, p
Demographics			
Age	24.2 ± 3.3, 18–28	23.0 ± 2.9, 18–30	*t* = 1.49, 0.14
Sex (Male: Female)	13:18	13:14	*χ* ^2^ = 0.04, 0.83
Full Scale IQ	118.4 ± 12.9, 92–146	113.8 ± 10.3, 97–137	*t* = 1.52, 0.13
Mental health concerns	25 (80.6%)	4 (14.8%)	OR = 0.05^a^, < 0.0001
Autistic traits			
SRS T‐Score	70.1 ± 11.8, 36–97	47.7 ± 6.2, 36–63	*t* = 9.23, < 0.0001
AQ Total Score	82.7 ± 13.1, 52–105	54.9 ± 10.2, 32–68	*t* = 9.09, < 0.0001
SPQ Hypersensitivity Total Score	25.7 ± 10.5, 7–47	11.6 ± 6.1, 2–23	*t* = 6.16, < 0.0001
SPQ Hyposensitivity Total Score	9.0 ± 4.9, 3–24	10.4 ± 4.9, 4–26	*t* = −1.05, 0.30
ADOS‐2 Comparison Score	6.4 ± 1.9, 2–9	—	—

^a^ OR: Odds Ratio (OR) from Fisher's exact test used when cell counts < 5; applicable for mental health concerns.

Those taking medications were required to have been stable on their regimen for at least 3 months prior to participation. In the autistic group, *N* = 21 (67.7%) participants reported current use of at least one psychotropic medication, compared to *N* = 2 (7.4%) in the non‐autistic group. The most commonly reported medication classes among autistic participants were stimulants (*N* = 12) and selective serotonin reuptake inhibitors (SSRIs; *N* = 10), including four participants taking both, whereas the two participants in the non‐autistic group were taking SSRIs only. Less frequently reported medications included antipsychotics (*N* = 4), antiepileptics (*N* = 3), SNRIs (*N* = 2), benzodiazepines (*N* = 1), and tricyclic antidepressants (*N* = 1).

The study was approved by the University of Washington Institutional Review Board, for which all participants provided informed consent and received compensation for their time.

### 2.2 Stimuli

Stimuli across the three experiments consisted of circular 6 Hz counterphase flickering checkerboards presented bilaterally on a gray background on luminance‐calibrated monitors. A flicker rate of 6 Hz was selected for two reasons. First, low‐frequency periodic stimulation provides high signal‐to‐noise in EEG and robustly drives steady‐state visual evoked responses in the occipital cortex (Norcia et al. 2015; Srinivasan et al. 2006). Second, flickering checkerboards are a well‐established stimulus in fMRI for eliciting reliable BOLD responses in early visual cortex (V1) and are widely used in standard visual localizer and retinotopic‐mapping approaches (Boynton et al. 1996; Engel 1997; Sereno et al. 1995). Each check was 1 degree of visual angle, and the total radius of the checkerboard was 6°, yielding a spatial frequency of 0.5 cycles/degree. This spatial frequency overlaps with the low end of spatial frequencies commonly used to estimate contrast sensitivity functions (e.g., Kéïta et al. 2014) and was chosen because low spatial frequencies elicit robust stimulus‐locked responses in EEG and reliable V1 responses in fMRI (Muthukumaraswamy and Singh 2008; Norcia et al. 2015). Each checkerboard had a Gaussian blur applied to 0.05° of visual angle. Each checkerboard was presented 8° to the left and to the right of a central fixation cross. The pairs of checkerboards were presented in a high (100%) and a low contrast (2%) condition. Across all experiments, the stimulus duration was 10 s and participants were instructed to fixate on the center of the screen. The psychophysics experiment included a target stimulus that matched the primary stimulus in all properties except for a reduced radius (3°) and contrast that was adaptively adjusted based on the participant's performance, as described below.

### 2.3 Psychophysics

#### 2.3.1 Apparatus

Stimuli were presented on a Windows computer using MATLAB's Psychtoolbox (Brainard 1997). Stimuli were presented using a 120 Hz calibrated monitor (Gamma = 1.56; luminance 128 cd/m^2^) at a viewing distance of 67 cm in a dimly lit room. Participants' responses were recorded using a keyboard.

#### 2.3.2 Experimental Procedure

**FIGURE 1:**
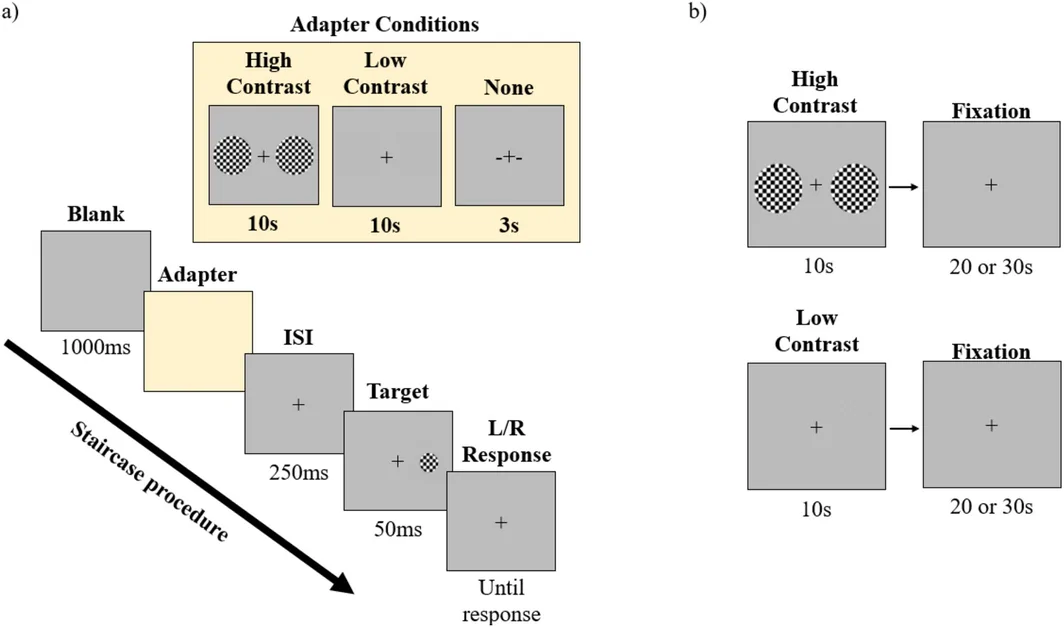
Experimental paradigms. (a) The psychophysical session used an adaptive staircase procedure to measure contrast detection thresholds under three adapter conditions: High contrast (100%), low contrast (2%) and no adapter. Each trial began with a blank screen followed by an adapter stimulus, interstimulus interval, and a target checkerboard. The target stimulus shared identical characteristics with the adapter stimulus except for a smaller radius. Participants were asked to maintain fixation on the center of the screen and respond to indicate the target location via left/right arrow presses. (b) The fMRI and EEG sessions employed a block design alternating 10 s of high or low contrast stimuli with resting fixation blocks. Resting blocks were either 20 s (for the EEG session) or 30 s (for the fMRI session). Contrast conditions were presented in separate runs, and runs alternated between high and low contrast conditions. The fMRI session consisted of three runs per condition, each containing six stimulus blocks, and the EEG session consisted of six runs per condition, each containing five stimulus blocks.

Participants engaged in a visual detection task; the target stimuli had identical properties to the visual‐response evoking flickering checkerboard, except for contrast properties that were adjusted following an adaptive staircase procedure based on an individual's performance. Participants were asked to respond whether they saw a target appear on the left or right of the screen via arrow keys. Target location (left or right) was randomized and balanced (50/50) independently of the staircase. Unlike a traditional staircase detection task, the experiment tested the effect of presenting the bilateral flickering checkerboards prior to the lateralized target presentation for each trial. We are calling the antecedent stimuli “adapters”, as they were meant to induce an adaptation effect. Participants underwent three versions of this detection task: one with no adapters, one with low (2%) contrast adapters, and one with high (100%) contrast adapters. On each trial, a blank gray pre‐stimuli screen was presented for 1000 ms, followed by a 10‐s flickering adapter screen, a 250 ms interstimulus interval, a 50 ms presentation of the target checkerboard, and a response screen that remained visible until the participant responded (as depicted in Figure 1a). A white fixation square was present from the adapter screen until response. Halfway through the session, participants were offered a brief break before continuing with the remaining half of the experiment. In the no adapters condition, each trial followed the same design except that the adaptation screen lasted 3 s and presented a white fixation square with two small black horizontal lines extending fixation.

Each participant completed two runs: an adapter run, which included four randomly ordered adaptive staircases (two with high contrast and two with low contrast adapters) interleaved throughout, and a no adapter run that included two staircases. Each staircase included 25 target trials, and each run included 14 catch trials to monitor participants' engagement. On each trial, the staircase selected a contrast based on the participant's cumulative responses, defined as the sequence of correct and incorrect target localization responses within that staircase. Binary accuracy values were used to update the estimated psychometric function and determine the next target contrast using the Psi‐method staircase from the Palamedes Toolbox (Prins and Kingdom 2018) with a Weibull psychometric function (gamma = 0.5; lapse rate = 0.04). The algorithm initially considered threshold priors between 0.15% and 15% and a slope of 0.25–0.25 (30 log‐spaced values). Target contrast levels that ranged from ~0.4% to 3% followed fine adjustments of ~0.4%, and contrast levels that ranged between 3% and 15% followed coarse adjustments of 1%. For each staircase, the next trial's contrast was set to the current mean threshold.

This paradigm was chosen to leverage contrast‐dependent adaptation effects in early visual cortex responsivity. When visual neurons are repeatedly activated by a stimulus, their response gain is attenuated, such that subsequent detection of similar visual input becomes more difficult. The inclusion of bilateral flickering checkerboards (“adapters”) allowed the manipulation of adaptation strength. High contrast adapters were expected to elicit a stronger neural visual response and consequently a greater reduction in response gain to subsequent stimuli, including the lateralized targets of the task. As a result of this reduced gain, participants were expected to exhibit decreased sensitivity to the targets, reflected as decreased detection performance. By embedding an adaptive staircase within this adaptation framework, the task provides a measure for an individual's visual sensitivity in the form of contrast detection thresholds, defined as the lowest contrast level detectable at a specified correctness probability (see Analyses below). Under increased adaptation, this paradigm predicts higher contrast detection thresholds.

#### 2.3.3 Analyses

For each staircase, trial‐by‐trial contrast detection threshold estimates and responses were saved. Analyses were performed in MATLAB using the Palamedes Toolbox. A Weibull function was fit to the aggregate proportions of correct counts at each contrast level using maximum‐likelihood; ultimately, the contrast detection threshold calculated equated to the contrast yielding ~82% correct responses. Staircases were identified as outliers if the average of the final five threshold estimates divided by the average of the first ten exceeded 0.8, meaning the participant needed almost just as much contrast at the end of the run as they did at the beginning. A total of seven outlier staircases were identified, all in the high contrast condition. Three participants (two autistic, one non‐autistic) had both high contrast staircases excluded and therefore did not have a psychophysical threshold estimate for that condition. One additional autistic participant had one outlier staircase excluded, and the remaining valid staircase estimate was retained for analysis. For all other participants, a single contrast threshold estimate was calculated for each condition by averaging the two staircase thresholds. These means were subsequently used for statistical analysis.

### 2.4 fMRI

#### 2.4.1 Apparatus

Scans were acquired using a Philips Ingenia Elition X 3T MRI system with a 32‐channel high‐resolution head coil. A T1‐weighted MPRAGE structural scan was collected at the start of the session with sagittal orientation (voxel size = 1:1:1 mm; acquisition matrix = 256 × 256 × 176; TR = 7.676 ms; TE = 3.5 ms, flip angle = 7°), followed by functional gradient‐echo echo‐planar imaging (EPI) runs with axial orientation (voxel size = 3:3:3.5 mm; acquisition matrix = 80 × 80 × 20; TR = 1 s; TE = 25 ms; flip angle = 79°). Stimuli were presented using Presentation software (Neurobehavioral Systems Inc.) in a 60 Hz projector.

#### 2.4.2 Experimental Procedure

Participants viewed stimuli projected onto a screen behind them using a mirror positioned above their eyes. Protective ear muffs were used to attenuate noise from the scanner. Functional scans were recorded while participants held fixation at the center of the screen and the bilateral checkerboards were presented. Participants were asked to press a button whenever they detected a color change in the fixation cross, from white to red. A total of 37 red fixation marks, presented for 66 ms each, were pseudorandomized per run; separated by at least 3.2 s from one another (an average of 1.5 events per 10‐s block). Performance in this task was of no interest for the main experimental design, but hit rate served as a measure of compliance. The fMRI session followed a block design in which blocks consisted of 10 s of stimulus presentation and 30 s of blank fixation, as shown in Figure 1b. Each participant completed six runs (three per contrast condition) with six blocks per run, resulting in 18 blocks per condition. For subsequent analyses, each stimulus block was treated as a single trial.

#### 2.4.3 Analyses

MRI data were preprocessed using fMRIPrep version 20.2.3 (Esteban et al. 2019) a Nipype based tool (Gorgolewski et al. 2011). For each participant, 1–4 T1‐weighted images were collected and underwent intensity non‐uniformity (INU) correction (N4BiasFieldCorrection v2.1.0; Tustison et al. 2010) and skull‐stripping (antsBrainExtraction.sh v2.1.0, OASIS template). A subject‐specific T1‐weighted map was generated from registration of their images using mri_robust_template (FreeSurfer v6.0.1; Dale et al. 1999). Brain surfaces were reconstructed using recon‐all (FreeSurfer v6.0.1) and the brain mask estimated previously was refined with a custom variation of the method to reconcile ANTs‐derived and FreeSurfer‐derived segmentations of the cortical gray‐matter of Mindboggle (Klein et al. 2017). Spatial normalization to the ICBM 152 Nonlinear Asymmetrical template version 2009c (Fonov et al. 2009) was performed through nonlinear registration with the antsRegistration tool (ANTs v2.1.0; Avants et al. 2008), using brain‐extracted versions of both T1‐weighted volume and template. Brain tissue segmentation of cerebrospinal fluid (CSF), white matter (WM), and gray matter (GM) was performed on the brain‐extracted T1‐weighted image using FAST (FSL v5.0.9; Jenkinson et al. 2002; Zhang et al. 2001).

Functional data preprocessing included the generation of a reference volume and a skull‐stripped version using a custom fMRIPrep method, correction for magnetic field inhomogeneity by estimating a B0‐nonuniformity map based on two EPI references with opposing phase‐encoding directions using 3dQwarp (Cox and Hyde 1997) (AFNI v16.2.07; Cox 1996), a slice timing correction using 3dTshift (AFNI), and motion correction using mcflirt (FSL v5.0.9). This was followed by co‐registration to the corresponding T1‐weighted image using boundary‐based registration (Greve and Fischl 2009) with 6° of freedom, using bbregister (FreeSurfer v6.0.1). Motion correction, susceptibility distortion correction, co‐registration of BOLD to T1‐weighted images, and normalization of the T1‐weighted image to MNI152NLin2009cAsym space were all concatenated and applied in a single interpolating step using antsApplyTransforms (ANTs v2.1.0, Lanczos interpolation).

Several possible confounds were generated in fMRIPrep: frame‐wise displacement (Power et al. 2014) was calculated for each functional run using Nipype, and low‐frequency regressors were generated using the DCT basis functions. For each run, the resulting 4D timeseries from fMRIPrep was then passed to the MATLAB toolbox *fmri_cleaning* (Mascali et al. 2021) for denoising, regressing out a first‐order polynomial trend to account for constant and linear trends, and the cosine confound regressors to account for low‐frequency signal drifts from the scanner or from physiological noise. After regression, the run mean was restored, and the residuals were written back to a NIfTI file for statistical modeling. No additional spatial smoothing was applied.

Regions of interest (ROIs) in early visual cortex (right and left hemisphere V1, V2, V3) for each participant were defined by the intersection of individual activation maps and a standard atlas, and were visually confirmed in BrainVoyager (Goebel et al. 2006). ROIs were identified from the first task run with the high contrast condition flickering checkerboard stimuli. A GLM was run to compare stimuli blocks with rest blocks, producing an activation map for each participant, reflecting general visual responsiveness. The activation maps were thresholded at *z* = 1.5 and then intersected with a custom probabilistic standard atlas of retinotopic areas (an intersection of retinotopic regions defined by Benson et al. 2012 and Rosenke et al. 2021). The top 20 most significant voxels (in functional space) within each ROI were used in the analysis.

The mean time courses were extracted from across the voxels in each ROI for each block. To compute to percent signal change, each time course was normalized by subtracting the mean value during baseline (4–0 s before stimulus onset), dividing by that mean, and multiplying by 100. For each block, we extracted data corresponding to 5 s before stimulus onset and 35 s after stimulus onset. Some blocks were excluded from analysis due to head motion or low task performance. Specifically, a block was excluded if framewise displacement (FD) exceeded 0.5 mm in any successive TR (Power et al. 2014; Siegel et al. 2014), up to and including 8 TRs before the stimulus block or 2 TRs after it. If more than half of the blocks of either contrast condition were excluded, or if a subject had a hit rate of < 60% in the central fixation task, the whole run was excluded. Participants with less than three runs were excluded from the analyses (only one autistic participant was excluded). The finalized dataset resulted in 6–18 blocks per condition for each participant (autistic group: M = 34.7, SD = 1.7; non‐autistic group: M = 32.5, SD = 5.9; *t*(55) = 1.97, *p* = 0.05). We further examined the possible contribution of performance differences to our neuroimaging findings by directly comparing the hit rate between groups. This analysis showed an overall high hit rate, and no differences between autistic (M = 0.96, SD = 0.06) and non‐autistic (M = 0.94, SD = 0.10) participants, *t*(55) = 1.25, *p* = 0.22.

We initially inspected an average time course across all participants to determine peak time windows of the BOLD response to apply for each contrast condition across participants. After visual inspection, we averaged the percent signal change from 10 to 12 s and from 7 to 9 s post stimulus onset for the high and low contrast conditions, respectively. To determine whether fMRI responses differed between hemispheres, we conducted a linear mixed‐effects ANOVA that tested the effect of hemisphere on fMRI response and controlled for contrast effects. We found no significant main effect of hemisphere (*F*(1,625) = 0.72, *p* = 0.40); therefore, we subsequently used averaged means across left and right hemispheres for analyses.

### 2.5 EEG

#### 2.5.1 Apparatus

A 128‐channel hydrocell sensor net was used to acquire electrical signals using the Geodesic EEG System 300 (Electrical Geodesics Inc.) with an acquisition sampling rate of 250 Hz. Signals were recorded using the CMS reference electrode and later re‐referenced to the common average across all sensors. Participants had the sensor net applied and the impedance for each electrode was adjusted as much as possible to 50 kΩ.

#### 2.5.2 Experimental Procedure

Stimuli presentation for the EEG session followed a similar experimental design to the fMRI session (shown in Figure 1b) and used a block design. Each stimulus block consisted of 10 s of stimulus presentation followed by 20 s of fixation. A run was defined as a continuous recording period containing 5 stimulus blocks of the same contrast condition (high or low contrast). The EEG session consisted of six runs per condition, resulting in 30 stimulus blocks per condition. Contrast conditions were presented in separate runs and alternated across the session, beginning with a high contrast run. Each block was treated as a single trial, time‐locked to stimulus onset and encompassing pre‐stimulus baseline and post‐stimulus activity (see Analyses section below). Stimuli were presented on a screen positioned 67 cm from the participant using Presentation software. Participants were seated at a table and used a chin rest to maintain a stable position throughout the experiment. The experiment was conducted in a room with lights turned off.

#### 2.5.3 Analyses

EEG data was processed using EEGLAB toolbox (Delorme and Makeig 2004) to downsample (to 100 Hz) and band‐pass filter (low frequency cutoff and transition band = 0.25 Hz/0.5 Hz; high frequency cutoff and transition band = 45 Hz/10 Hz) the time series. Trials were segmented beginning 5 s before visual stimulus onset and lasting 30 s. The time series was inspected for bad channels, electrodes with poor scalp contact, or other artifacts. Bad channels, identified by consensus between three experts after visual inspection, were excluded from analysis, and when needed, replaced by spatial interpolation of signals from adjacent electrodes. Remaining signals were processed using independent component analysis (ICA) and descriptive statistics to estimate probabilities of a component's categorical significance across seven possible categories (Pion‐Tonachini et al. 2019). Components with probabilities greater than 0.8 in the “eye” category were inspected and removed. The remaining components were remixed to create the final segment of data. Three participants were excluded from further analysis due to (1) a large number of bad channels (only one non‐autistic participant had > 20% bad channels; across groups, the average number of bad channels identified per participant was low: autistic M = 5.5, SD = 0.96; non‐autistic M = 9.0, SD = 1.7), (2) persistent and repetitive blinking across all trials (only one autistic participant), and (3) an insufficient number of clean trials (< 10) per condition at location Oz using a ±75 mV rejection threshold (only one non‐autistic participant with 2/30 clean trials). Across groups, the average number of rejected trials at Oz was low for both the high contrast (autistic M = 2.8, SD = 4.7; non‐autistic M = 2.5, SD = 3.2) and low contrast (autistic M = 2.7, SD = 4.8; non‐autistic M = 2.4, SD = 3.9) conditions. After artifact rejection and participant exclusion, the same final sample of participants was retained across all conditions.

**FIGURE 2:**
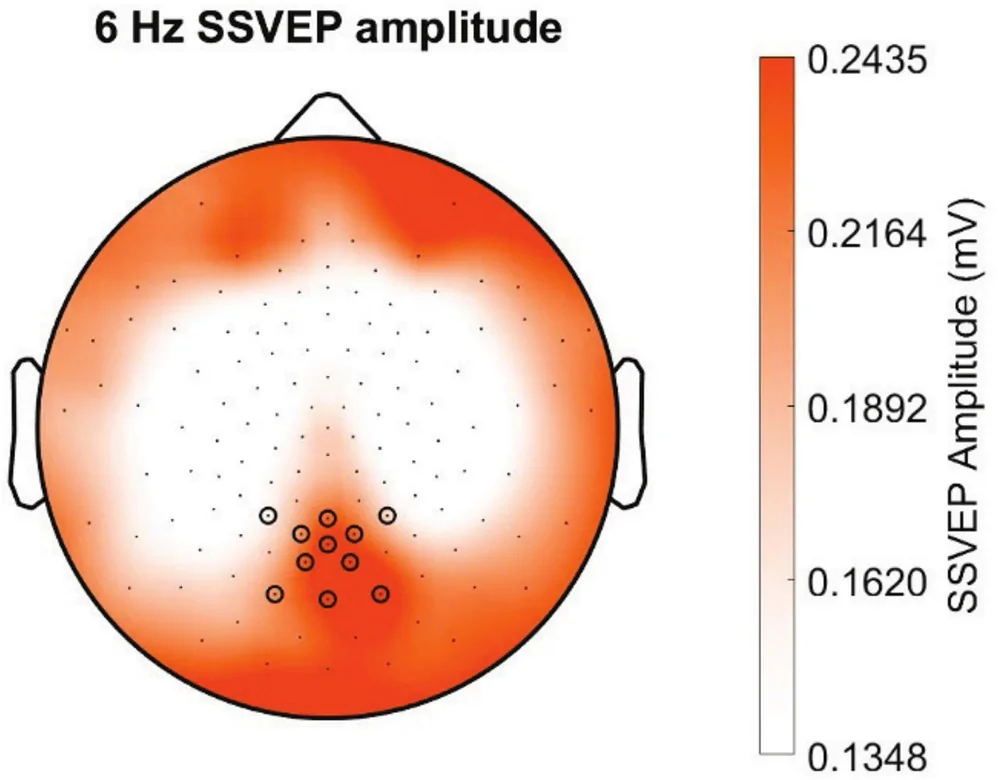
Methodological analysis. Grand‐average scalp distribution of 6 Hz SSVEP amplitude, averaged across all participants and contrast conditions during the last 5 s of the sustained stimulus presentation window. An average signal was calculated from the highlighted electrodes for subsequent analyses: *O1*, *O2*, *Oz*, *71*, *76*, *PO3*, *PO4*, *Pz*, *Poz*, *P1*, and *P2*.

We analyzed the EEG data using SSVEP analyses to capture aspects of neural responsiveness as SSVEPs reflect ongoing oscillatory responses at the driving frequency, providing a sensitive measure of sustained visual processing during stimulus presentation. Data was extracted from the final 5 s of stimulus presentation, corresponding to the steady‐state portion of the response after stimulus‐onset. For each participant and condition, data was first averaged across trials in the time domain to enhance stimulus‐locked activity, and a spectral analysis was performed. Artifact‐rejected trials were identified on an electrode‐by‐trial basis and coded as missing values. Trial averaging was then performed separately for each electrode using only non‐missing trials, such that the number of retained trials could vary across electrodes. A fast Fourier transform was applied to the trial‐averaged EEG time series for each electrode after downsampling to 100 Hz. The FFT was computed along the time dimension using MATLAB's *fft* function, without zero‐padding or windowing. The resulting amplitudes were normalized by the number of time points and only the positive frequency components (0 to Nyquist frequency) were retained. Frequency resolution was determined by the length of the analyzed epoch (Fs/N). The resulting FFT amplitudes were then averaged across a preselected group of occipital and parietal electrodes (70(*O1*), 83(*O2*), 75(*Oz*), 71, 76, 67(*PO3*), 77(*PO4*), 62(*Pz*), 72(*Poz*), 60(*P1*), and 85(*P2*), see Figure 2b). Electrodes were selected a priori based on their posterior scalp location over the visual cortex. Mean SSVEP amplitudes were calculated as the maximum amplitude within ±0.5 Hz of the foundational frequency (6 Hz).

### 2.6 Sensory Questionnaires

Participants completed the Sensory Perception Quotient (SPQ, Tavassoli et al. 2014) questionnaire as part of a larger battery of behavioral assessments. The SPQ is a 92‐item self‐report that assesses sensory sensitivities. We extracted scores for the hypersensitivity and hyposensitivity subscales. There was missing SPQ data (questionnaire not completed) for one autistic participant and three non‐autistic participants.

### 2.7 Statistical Analyses

Between‐group analysis investigated the effects of contrast condition, diagnostic group, and their interaction using linear mixed‐effects models holding subject as a random intercept to account for repeated measures across experiments. The contrast condition factor had two levels (high and low contrast) for the fMRI and EEG experiments, and three levels for the psychophysics experiment (with the additional accounting for the no adapter condition).

Exploratory within‐group analyses aimed to investigate the congruency across all measures. This analysis aimed to test how an individual's response magnitude in one measure correlated with the response magnitude in another measure. Within‐group correlations were run to test correlations across measures per condition. Significant associations were followed up with appropriate linear regression models. Additionally, we examined associations between experimental measures and self‐reported hyper‐ and hyposensitivity scores using correlational analyses. When appropriate, we further assessed quadrant membership with Fisher's exact test to account for small sample sizes in each quadrant.

## 3 Results

### 3.1 Between‐Subject Analysis

We tested visual responsivity to a bilaterally presented counterphase flickering circular checkerboard and found that contrast condition was a significant modulator of visual responses across psychophysics, fMRI, and EEG experiments. Low contrast stimuli elicited reliable SSVEP and BOLD responses above baseline (see Supplemental Analysis 1). Groups were comparable across most of the measured responses, with the only exception being SSVEP amplitudes, for which autistic individuals had significantly higher amplitudes than non‐autistic participants.

#### 3.1.1 Psychophysical Contrast Detection Thresholds

**FIGURE 3:**
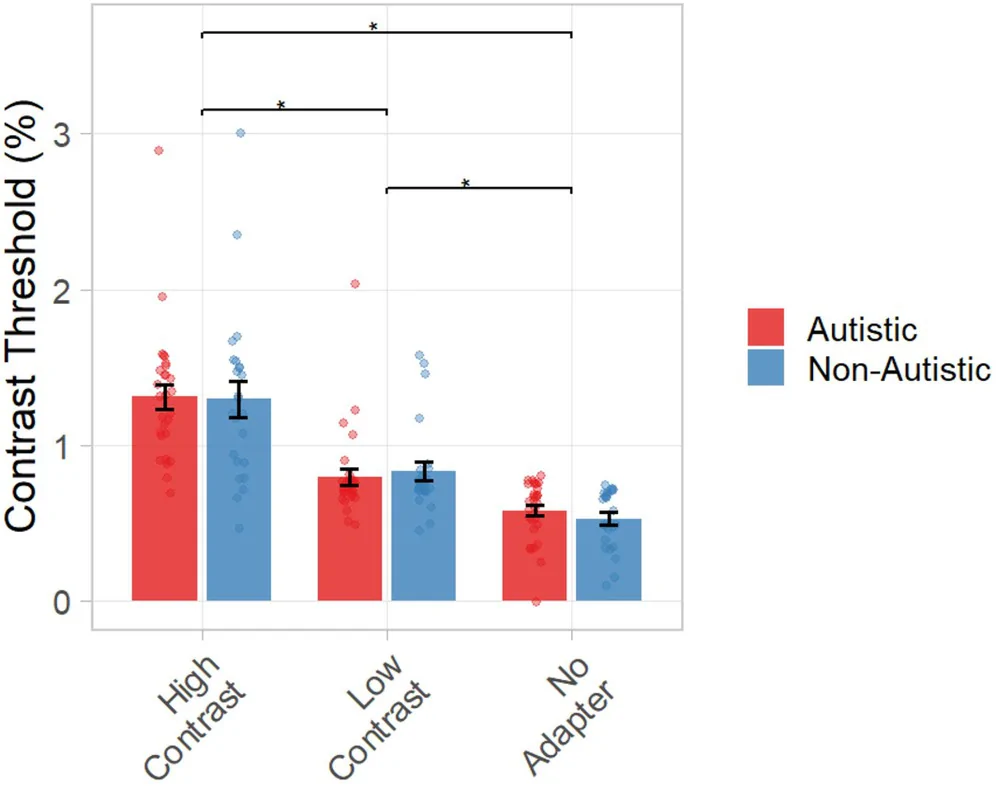
Contrast detection thresholds. Mean contrast detection thresholds (±SEM) for non‐autistic and autistic participants across adapter conditions (high contrast, low contrast, and non‐adapter). Individual data points are overlaid on the barchart, and significant pairwise differences between adapter conditions are indicated by asterisks (*p < 0.05).

A linear mixed‐effects model examining the effect of group, adapter condition, and their interaction on contrast detection thresholds indicated that adapter condition had a significant effect, *F*(2, 101.91) = 70.50, *p* < 0.0001, *ηp* ^2^ = 0.58 (Figure 3). The effect of group on contrast detection threshold was not significant, *F*(1, 50.25) = 0.04, *p* = 0.85, *ηp* ^2^ < 0.001, and neither was their interaction, *F*(2, 101.91) = 0.24, *p* = 0.78, *ηp* ^2^ = 0.005. Following upon the significant effect of the adapter condition, pairwise comparisons using Tukey's test indicated significant differences across all tested pairs of conditions. Specifically, contrast detection thresholds were significantly higher in the high contrast condition compared to the low contrast (*t*(105) = 7.67, *p* < 0.0001, Cohen's *d* = 1.49) and the no adapter (*t*(105) = 11.72, *p* < 0.0001, Cohen's *d* = 2.28) conditions. Similarly, contrast detection thresholds were significantly higher in the low contrast condition than in the no adapter condition (*t*(103) = 4.11, *p* = 0.0002, Cohen's *d* = 0.79). The robust effect of adapter condition is consistent with our hypothesis that visual responsivity, particularly under higher contrast conditions, would elevate visual adaptation and therefore increase contrast detection thresholds due to reduced sensitivity to subsequent targets.

#### 3.1.2 fMRI Visual Activation

**FIGURE 4:**
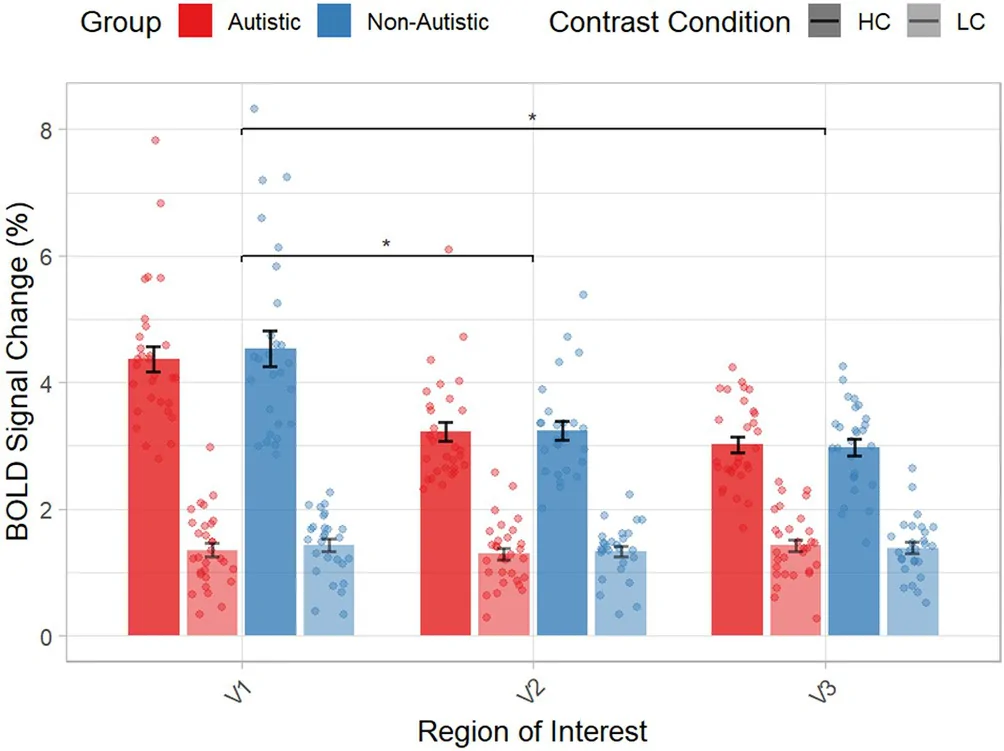
BOLD signal change in the early visual cortex. Mean percent signal change (±SEM) across early visual ROIs (V1, V2, V3) for non‐autistic and autistic participants for high contrast (HC) and low contrast (LC) conditions. Individual data points are overlaid on the barchart, and significant pairwise differences between ROIs are indicated by asterisks (*p < 0.05). A significant main effect of contrast (HC > LC) was observed across all ROIs, although not explicitly annotated for clarity.

A linear mixed‐effects model testing the contribution of group, contrast, ROI, and the interaction between group and contrast revealed significant main effects of contrast, *F*(1, 281) = 791.15, *p* < 0.0001, *ηp* ^2^ = 0.74, and ROI, *F*(2, 281) = 34.36, *p* < 0.0001, *ηp* ^2^ = 0.20 (Figure 4). The high contrast condition elicited a significantly greater signal change (M = 3.56, SD = 1.14) than the low contrast condition (M = 1.37, SD = 0.51). Following up on the significant main effect of ROI, Tukey's HSD post hoc tests indicated that V1 had significantly greater response changes (M = 2.92, SD = 1.83) compared to both V2 (M = 2.27, SD = 1.16; *t*(281) = 6.81, *p* < 0.0001, Cohen's *d* = 0.90) and V3 (M = 2.20, SD = 1.00; *t*(281) = 7.50, *p* < 0.0001, Cohen's *d* = 0.99); while no significant difference was observed between V2 and V3, *t*(281) = 0.69, *p* = 0.77, Cohen's *d* = 0.09. The stronger response in V1 compared to V2 and V3 is consistent with the hierarchical organization of the visual cortex where V1 typically shows the largest BOLD responses to simple contrast stimuli. Contrastingly, belonging to the autistic (M = 2.45, SD = 1.38) or non‐autistic (M = 2.48, SD = 1.45) group did not have an effect on fMRI responses, *F*(1, 55) = 0.08, *p* = 0.78, *ηp* ^2^ = 0.001.

#### 3.1.3 SSVEPs

**FIGURE 5:**
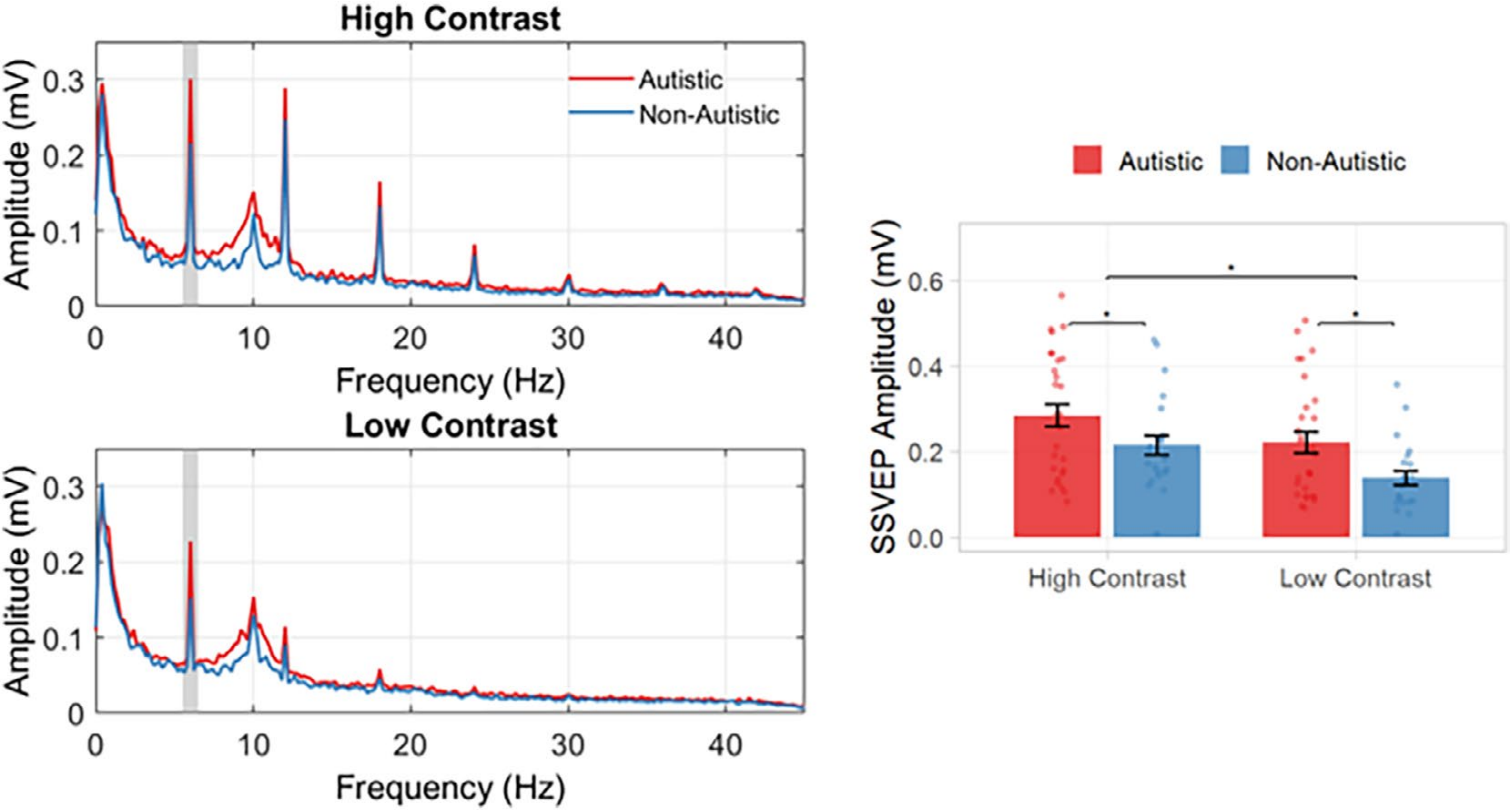
SSVEP amplitude responses. SSVEP responses and group differences across contrast conditions. Left: Grand‐average frequency spectra of SSVEPs averaged across participants for the non‐autistic and autistic groups for each contrast condition. Peaks at 6 Hz and its respective harmonics reflect neural entrainment to the flickering stimulus. Right: Mean SSVEP responses (±SEM) for each group and condition at the fundamental frequency (6 Hz), with individual data points overlaid. Brackets with asterisks (*) indicate significant differences (*p* < 0.05) between groups (autistic > non‐autistic) and contrast conditions (high contrast > low contrast).

A linear mixed‐effects model examining the effect of group, contrast condition, and their interaction on SSVEP amplitude indicated that both group (*F*(1, 50) = 4.65, *p* = 0.035, *ηp* ^2^ = 0.09) and contrast condition (*F*(1, 466) = 59.51, *p* < 0.0001, *ηp* ^2^ = 0.11) had a significant effect (Figure 5), and there was no significant interaction between the two, *F*(1, 466) = 0.09, *p* = 0.76, *ηp* ^2^ < 0.001. Following upon significant main effects, the high contrast condition elicited significantly higher SSVEP amplitudes than the low contrast condition (diff = 0.07, Cohen's *d* = 0.68), and the autistic group had higher SSVEP responses than the non‐autistic group (diff = 0.03, Cohen's *d* = 0.27). Exploratory within‐group analyses indicated that SSVEP amplitude did not differ as a function of SSRI or stimulant use within the autistic group, and medication status did not alter contrast‐related modulation of SSVEP responses (see Supplemental Figure 1).

### 3.2 Within‐Subject Analysis

Given the results above, our analysis focused on examining associations between SSVEP amplitudes and all other neural measures and behavioral scores.

#### 3.2.1 SSVEPs And Contrast Detection Thresholds

**FIGURE 6:**
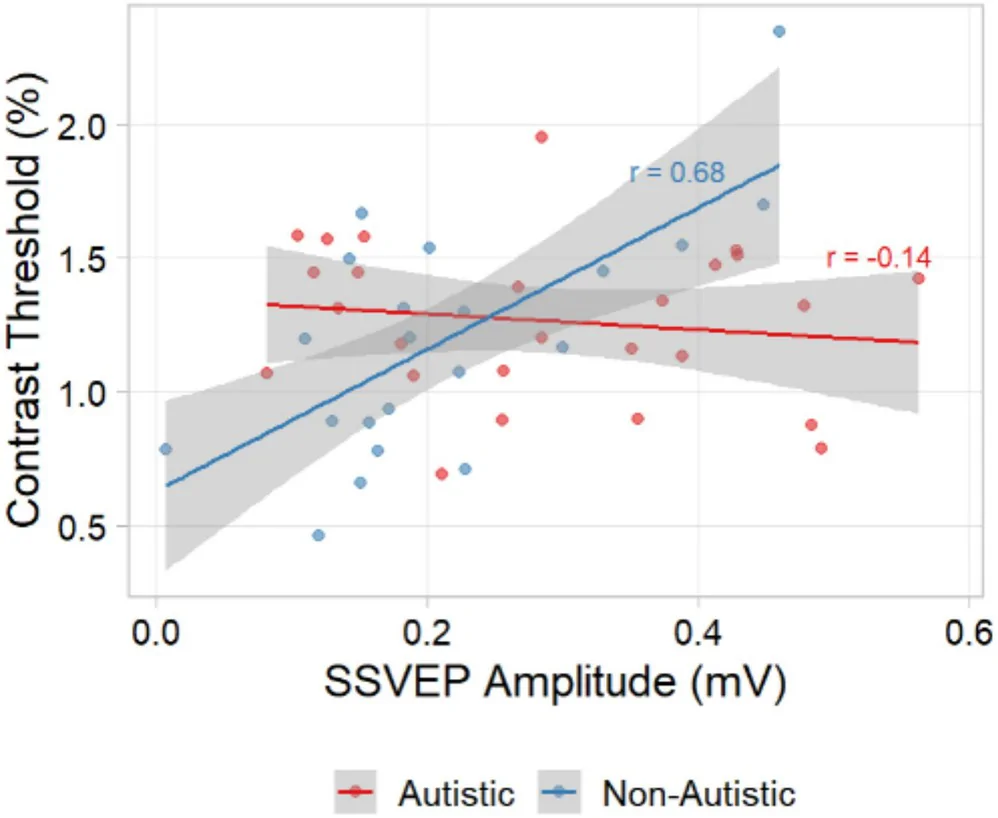
Associations between SSVEP amplitude and contrast detection thresholds. Mean contrast detection threshold and SSVEP amplitude per participant, with shaded regions indicating 95% confidence intervals for linear fit models for both non‐autistic (*r* = 0.68) and autistic (*r* = 0.14) groups.

A linear regression model was used to examine the association of SSVEP amplitudes and contrast detection thresholds (Figure 6) and whether this relationship differed by group for each condition. The analysis revealed no significant associations in the low contrast condition. In the high contrast condition, there was a significant main effect of SSVEP amplitude on contrast thresholds, *F*(1, 43) = 4.53, *p* = 0.039, and a significant interaction effect between SSVEP amplitude and group when predicting contrast thresholds, *F*(1, 43) = 15.02, *p* < 0.001. A follow‐up analysis showed that for the non‐autistic group, higher SSVEP amplitudes were associated with higher contrast detection thresholds (*r* = 0.68), whereas for the autistic group, there was no significant association (*r* = −0.14). The difference in slopes between groups was statistically significant, *t*(43) = −3.88, *p* < 0.001.

#### 3.2.2 SSVEPs and BOLD

**FIGURE 7:**
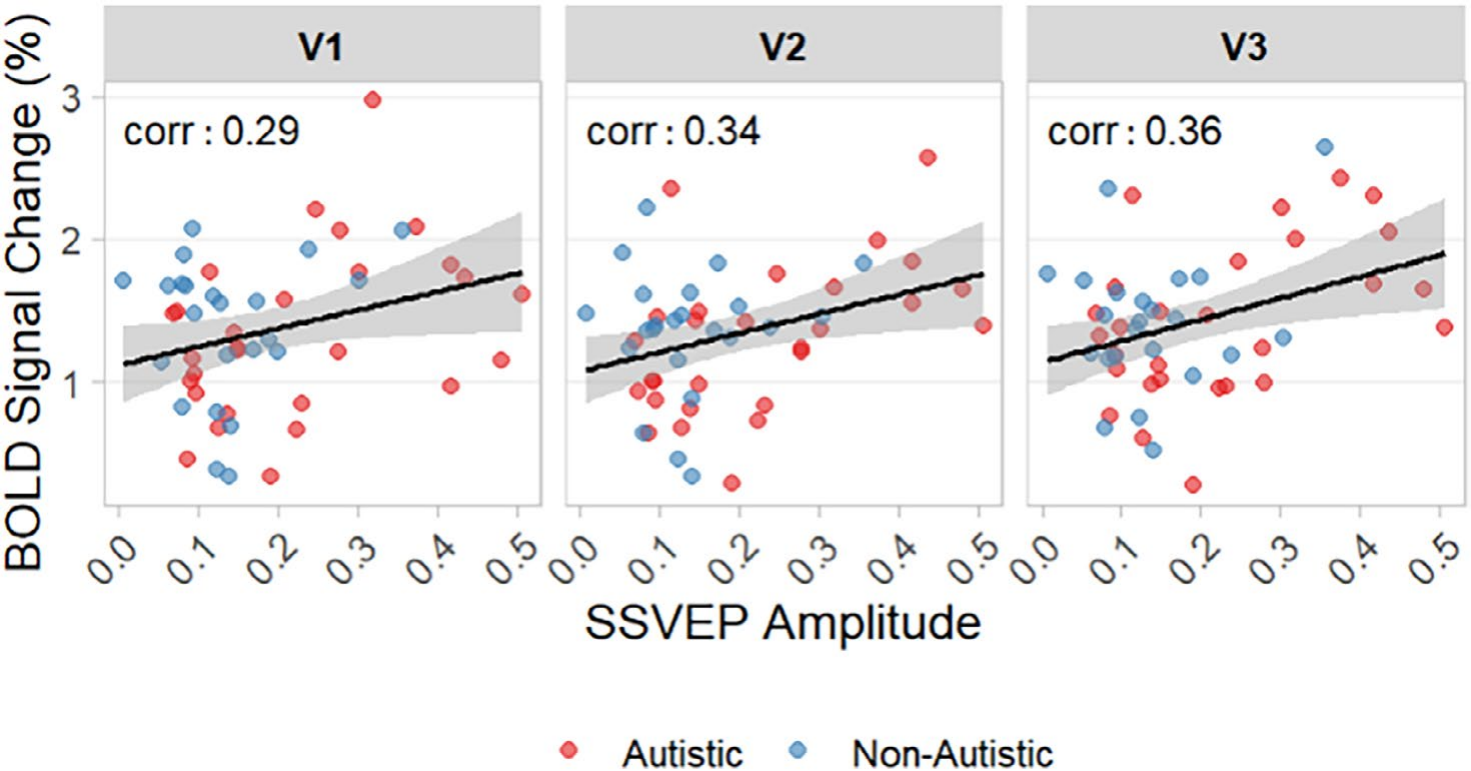
Association between SSVEP amplitude and BOLD signal change. Mean BOLD (%) signal change and SSVEP amplitude per participant. Shaded regions indicate 95% confidence intervals for linear fit models across V1 (*r* = 0.29), V2 (*r* = 0.34), and V3 (*r* = 0.36).

We used linear mixed‐effects models, for each condition, to test the association between SSVEP amplitudes and BOLD activity (Figure 7), controlling for ROI, group, and repeated measures. Only the low contrast condition model found SSVEP amplitudes to be a significant predictor of BOLD signal, *F*(1, 48) = 8.89, *p* = 0.004. Specifically, we observed positive correlations between SSVEP amplitudes and BOLD activity across V1 (*r* = 0.29, *p* = 0.04), V2 (*r* = 0.34, *p* = 0.02), and V3 (*r* = 0.36, *p* = 0.01).

#### 3.2.3 SSVEPs and Sensory Sensitivity

**FIGURE 8:**
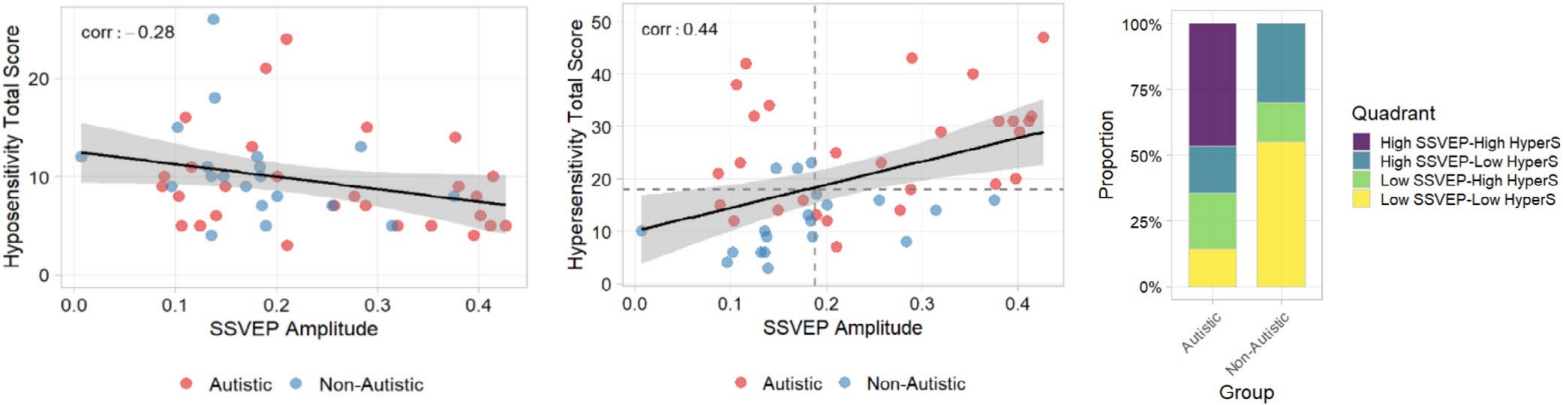
Associations between SSVEP amplitude and sensory sensitivity scores on the SPQ. Associations between SSVEP amplitude and hypo‐ and hypersensitivity total scores on the SPQ across non‐autistic and autistic participants. Left: Scatterplot showing the correlation between SSVEP amplitude and hyposensitivity scores (*r* = −0.28), with linear fit line and 95% confidence intervals for each group. Center: Scatterplot showing the correlation between SSVEP amplitude and hypersensitivity scores (*r* = 0.44), with linear fit line and 95% confidence interval. Dashed lines indicate the median split across SSVEP amplitude values and hypersensitivity scores (used to define quadrants). Right: Stacked bar plots showing the proportion of individuals falling into each quadrant of the SSVEP‐hypersensitivity distribution by diagnostic group.

A linear regression analysis was conducted to examine the relationship between an individual's SSVEP amplitude (averaged across high and low contrast conditions) and sensory hyper‐ and hyposensitivity (Figure 8), as measured by the SPQ. Results revealed that SSVEP amplitudes had a significant positive association with hypersensitivity (*r* = 0.44, *p* = 0.002), and a non‐significant negative association with hyposensitivity (*r* = −0.28, *p* = 0.05).

To evaluate the combined contribution of SSVEP amplitude and hypersensitivity scores in the classification of individuals, participants were grouped into one of four quadrants defined by the sample's medians: (1) high SSVEP and high hypersensitivity, (2) high SSVEP and low hypersensitivity, (3) low SSVEP and high hypersensitivity, and (4) low SSVEP and low hypersensitivity. A Fisher's exact test revealed a statistically significant association between group and quadrant membership, suggesting that combined SSVEP and hypersensitivity profiles effectively differentiated between autistic and non‐autistic individuals. Notably, only autistic individuals were observed in the high SSVEP and high hypersensitivity quadrant, which represented almost 50% of the autistic sample. In contrast, the majority of non‐autistic individuals were located in the low SSVEP and low hypersensitivity quadrant.

## 4 Discussion

This study aimed to investigate group differences in behavioral and neural responses to periodic visual stimuli in autistic and non‐autistic adults. Prior research examining neural excitability has yielded mixed results, possibly due to the variability in methods with differing stimuli and experimental paradigms. To address this, the current study leveraged a consistent visual stimulus, 6 Hz counterphase flickering checkerboards presented bilaterally, across three independent experiments using psychophysics, fMRI, and EEG tasks to elucidate the neural substrates involved in visual responsivity in autistic and non‐autistic individuals. This design allowed us to examine how distinct measurement domains index complementary consequences of the same rhythmic visual stimulus, rather than assuming equivalence across outcome measures.

As we expected, increased visual stimulation (i.e., presentation of a higher contrast stimulus) evoked larger brain responses across participants. The high contrast condition, across the three experiments, resulted in higher fMRI activity and EEG responses compared to the low contrast condition, consistent with previous literature (Boynton et al. 1996; Chen et al. 1990; Souza et al. 2007). In the psychophysics paradigm, we observed an expected adaptation effect: the presentation of a stimulus (i.e., adapter) reduced visual sensitivity to the subsequent presentation of an equivalent stimulus (i.e., target). This behavioral measure captured the downstream perceptual effects of prior periodic stimulation, rather than the direct response to the flickering stimulus. This adaptation was modulated by adapter contrast; a higher contrast adapter, eliciting greater neural activity, produced a stronger adaptation effect, resulting in higher contrast detection thresholds.

We hypothesized that autistic individuals would show heightened neural responsivity to periodic stimuli—particularly in measures sensitive to stimulus‐locked synchronization—and, consequently, altered perceptual sensitivity following adaptation. Consistent with this prediction, group differences were observed in SSVEP amplitudes, with autistic individuals exhibiting significantly higher amplitudes across contrast conditions. Group differences were not observed in BOLD responses or contrast detection thresholds. Notably, SSVEP amplitudes were related to both BOLD responses under certain conditions and to self‐reported sensory hypersensitivity, suggesting that individual differences in frequency‐locked neural responses capture variation in both visual cortical activity and subjective sensory experiences.

The present SSVEP findings suggest that autistic adults showed heightened stimulus‐locked synchronization to periodic visual input. Larger SSVEP amplitudes indicate stronger population‐level entrainment to the temporal structure of the stimulus, specifically the coordinated visual cortical response to repeated contrast reversals. In this context, entrainment can be understood as rhythmic modulation of visual cortical responsiveness. Neural oscillations are thought to reflect alternating periods of relatively higher and lower excitability, such that the phase of an oscillation influences the likelihood and gain of neural responses to incoming input (Lakatos et al. 2005; Schroeder and Lakatos 2009). When visual input is presented rhythmically, as it is here, neural activity can become phase‐locked to the stimulus rhythm, resulting in periods in which visual cortical populations are more responsive to recurring input at the stimulus frequency (Lakatos et al. 2008; Schroeder and Lakatos 2009). Thus, a larger SSVEP at 6 Hz can be interpreted as evidence of a larger or more coherent population‐level response that is synchronized to the temporal structure of the stimulus.

An important consideration is whether the present findings are specific to the 6 Hz stimulus frequency used in this study or reflect a broader difference in visual responsivity across temporal frequencies. High frequency flicker rates are commonly used to probe rapid temporal processing, including responses near or above the perceptual limits of flicker detections, whereas low frequency SSVEP paradigms preserve more of the response structure associated with individual stimulus events and are better suited for examining synchronization to slower, visible changes in visual input (Herrmann 2001; Norcia et al. 2015). The 6 Hz frequency falls within the low‐frequency range and therefore probes the visual system's ability to track and sustain responses to rhythmic changes in contrast. This is relevant to general visual processing because visual perception depends on continuously updating information as sensory input changes over time. If the present effect is specific to 6 Hz, it may indicate that autistic individuals differ in entrainment to relatively slow rhythmic visual input. This is of clinical relevance because sensory differences in autistic individuals commonly include heightened responses to visual features such as lights and movement, and flickering or time‐varying visual input can evoke discomfort in ways that depend on its temporal structure (Tavassoli et al. 2014; Yoshimoto et al. 2017).

Prior SSVEP work has shown that visual response group differences vary across temporal frequencies and are not uniform across stimuli. At lower frequencies, Takarae et al. (2016) reported larger contrast‐dependent responses to 3.76 Hz gratings in autistic participants, particularly at higher contrasts. Vilidaite et al. (2018) reported frequency‐ and harmonic‐specific SSVEP group differences: autistic adults and adults with high autistic traits showed relatively intact or slightly larger first‐harmonic responses to a 7 Hz stimulus but reduced second‐harmonic responses, whereas autistic children showed reduced first‐harmonic responses to a 5.12 Hz stimulus. Studies using faster flicker rates, such as 12.5 Hz, have found reduced responses in low‐contrast conditions and increased neural noise in autistic children (Weinger et al. 2014). Related work using a 5 Hz visual frequency stimulus also found reduced first harmonic responses at low contrast in autistic children (Norcia et al. 2021), supporting the idea that group differences may depend on temporal frequency, contrast, response harmonic, and developmental stage. The present results are consistent with the broader literature showing frequency‐ and condition‐specific visual response differences in autism and should not be interpreted as evidence of a generalized increase in SSVEP responsivity across all temporal frequencies. Future studies that directly manipulate stimulus temporal frequency should take these variables into account.

One possible interpretation of the selective group difference in SSVEP amplitudes is that autistic and non‐autistic adults differ specifically in the visual system's ability to synchronize with and sustain oscillatory activity at the stimulus frequency, rather than in visual responsivity across all measurement modalities. This would indicate that temporal processing of visual information may differ in autism. SSVEP amplitudes reflect strength of neural entrainment to periodic visual input (Norcia et al. 2015; Prado‐Gutiérrez et al. 2019), which depends on coordinated excitatory and inhibitory processes. Elevated SSVEPs in autistic adults therefore likely reflect stronger or more sustained stimulus‐locked neural responses, rather than a nonspecific increase in neural excitability. This interpretation is consistent with the observation that group differences were detected in SSVEP amplitudes but not in fMRI responses, as the latter primarily capture integrated response magnitude rather than sustained phase‐locked synchronization. This fits with past research that has reported differences in visual temporal processing in autistic samples (Chen et al. 2012; Foss‐Feig et al. 2010; Jemel et al. 2010; Manning et al. 2015; Robertson et al. 2014; Thompson et al. 2015).

An alternative possibility is that the selective detection of group differences in SSVEPs reflects differences in sensitivity across measurement modalities. Periodic flickering stimuli are specifically optimized to elicit SSVEPs, which benefit from high signal‐to‐noise ratios due to averaging across cycles (Norcia et al. 2015), making them uniquely sensitive to group‐level differences. In contrast, BOLD responses are transient, happening in a constrained amount of time after stimulus onset and, therefore, susceptible to trial‐to‐trial variability (Kappenman and Luck 2012; Logothetis et al. 2001; Norcia et al. 2015); potentially limiting their sensitivity during sustained rhythmic visual input. Furthermore, the slow temporal resolution of BOLD activity may overlook temporal differences (Logothetis et al. 2001). Consistent with this interpretation, we performed split‐half reliability analyses that demonstrated higher measurement stability for SSVEP amplitudes than for fMRI responses—particularly in the low contrast condition—supporting the idea that method‐specific sensitivity differences contributed to the selective detection of group effects (see Supplemental Analysis 2). Therefore, the absence of group differences in these measures should not be interpreted as evidence that visual processing differences cannot be detected using fMRI or behavioral paradigms more generally, but rather that the present stimulus was best matched to SSVEP‐based measures.

Within‐subject analyses provided some evidence for convergence across neural measures. Specifically, larger SSVEPs were associated with larger BOLD responses in the low contrast condition, suggesting that frequency‐locked EEG responses captured individual differences that were also reflected in visual cortical hemodynamic activity. Prior work has shown that SSVEP and BOLD responses can covary across different frequencies of visual stimulation, particularly within the occipital visual cortex (Singh et al. 2003). Therefore, the present finding is consistent with prior evidence that SSVEP amplitudes can reflect individual differences in visually evoked cortical activity, although SSVEP and BOLD signals should not be interpreted as indexing identical neural mechanisms. However, this association was not observed in the high contrast condition, which may reflect a saturation effect at higher contrast and suggests that the extent to which neural signals across methods relate to each other may be most detectable when stimulus drive is not near ceiling.

A notable group interaction emerged when assessing the relationship between SSVEP amplitudes and contrast detection thresholds across conditions, specifically at high contrast. Among non‐autistic participants, higher SSVEP amplitudes were associated with greater adaptation effects (e.g., increased contrast thresholds), consistent with sensory adaptation mechanisms (Greenlee and Heitger 1988). In contrast, this relationship was not present in the autistic group. This dissociation suggests that neural synchronization and perceptual adaptation may be less tightly coupled in autistic adults, particularly under high contrast conditions. Such dissociation may reflect differences in how sustained neural responses influence subsequent perceptual sensitivity.

We also found that individuals who exhibited larger SSVEPs also reported higher levels of hypersensitivity. This association provides a clinically relevant link between heightened neural entrainment to periodic visual stimulation and individual differences in sensory experiences. One possibility is that stronger or more persistent neural entrainment to flickering input increases the perceptual salience or aversiveness of visual stimuli, contributing to hypersensitivity. Alternatively, heightened synchronization may enhance certain aspects of visual processing at the cost of flexibility or suppression of irrelevant input, which could manifest as sensory overload in complex environments.

These findings are consistent with a small literature linking early visual neural measures to sensory traits in autism. For example, Takarae et al. (2016) found that steeper SSVEP contrast‐response slopes were associated with greater parent‐reported sensory difficulties, suggesting that heightened visual responsivity may be clinically relevant for sensory differences. Other work has similarly linked ERP responses, including P1 amplitudes and SSVEP response properties to visual hyperresponsiveness (Shuffrey et al. 2018) and autistic traits (Jackson et al. 2013; Vilidaite et al. 2018). However, these associations are not consistent across the literature. For example, Sapey‐Triomphe et al. (2023) found elevated self‐reported sensory sensitivity in autistic adults, but no group differences in neural visual thresholds and no significant associations across these measures. Given the mixed literature, one possibility is that associations between early visual neural responses and sensory traits are most evident in specific subgroups rather than across all autistic individuals.

Consistent with this possibility, the association between SSVEP amplitudes and hypersensitivity was further illustrated when individual neural and sensory scores were considered together. Almost half of all autistic participants fell in the quadrant representing both high SSVEP and high hypersensitivity. Notably, this quadrant had no non‐autistic participants present. Conversely, most non‐autistic individuals were located in the low SSVEP and low hypersensitivity quadrant. These findings underscore the added value of incorporating neurobiological measures in the characterization of groups. Given that the autistic group was over‐represented with co‐occurring ADHD, we visually examined the distribution of ADHD diagnoses across the plot (see Supplemental Figure 2). Individuals with ADHD were present across all four quadrants, suggesting that the observed clustering was not solely driven by ADHD diagnosis.

Some limitations should be considered when interpreting this study's findings. First, the modest sample size limits statistical power and constrains the inferences that can be drawn, particularly when investigating a highly heterogeneous group such as the autistic population. Although sample sizes were comparable to prior multi‐method neuroimaging studies, the autistic population is characterized by substantial inter‐individual variability in sensory processing, neural dynamics, and clinical presentation (Lenroot and Yeung 2013; Lombardo et al. 2019), which likely attenuates group‐mean effects and increases the risk of type I error. Sensitivity analyses indicated that the present sample sizes were sufficient to detect medium‐to‐large between‐group effects (~0.76–0.80 across methods, see Supplementary Analysis 3), but underpowered for small group differences. Therefore, null group effects in behavioral and hemodynamic measures and positive group effects in electrophysiological measures should be interpreted cautiously and warrant investigation in larger samples. At the same time, the study demonstrated strong sensitivity to within‐subject manipulations, as reflected by large contrast‐dependent effects across the three experiments. Together, these considerations underscore the importance of future studies with larger samples to more fully characterize small‐magnitude effects, individual variability, and potential subgroups within the autistic population.

Second, while we examined hyper‐ and hyposensitivity using a self‐report measure, these scores may not fully capture the complexity or multidimensional nature of sensory experiences in autism (e.g., variability across modalities, context, or over time), potentially limiting our ability to detect their relationship to neural responsiveness. An additional limitation of the study is the use of only two stimulus contrast levels and a single low spatial frequency. While the design enabled a comparison across modalities, it limits the ability to fully characterize the contrast‐response function across spatial frequency ranges. In particular, responses for the high contrast condition may have approached saturation, which could reduce sensitivity to group differences that might be stronger at intermediate contrast levels. Future studies employing a broader range of contrast and spatial frequency levels would contribute to a detailed assessment of contrast‐ and spatial‐frequency‐dependent neural responsivity. Lastly, the study's sample was exclusively adults, and thus does not capture developmental trajectories of sensory processing differences. It is possible that compensatory attentional mechanisms that develop across the lifespan could potentially mask group differences in early sensory processing.

Overall, these findings point to measure‐specific differences in visual responsivity in autism, with significant group differences emerging in SSVEP amplitudes. These SSVEP differences were not isolated findings. SSVEP amplitudes were associated with BOLD responses in the low contrast condition and with individual differences in sensory hypersensitivity. Specifically, autistic individuals showed heightened neural synchronization during periodic visual input, as indexed by SSVEPs, in comparison to non‐autistic individuals. These group differences were not observed in hemodynamic and behavioral responses under the present experimental conditions. These results suggest that frequency‐locked EEG responses may be particularly sensitive to group differences in visual responsivity, especially in relation to individual differences in sensory hypersensitivity.

## 5 Conclusions

This study leveraged a consistent visual stimulus across psychophysics, fMRI, and EEG tasks to explore sensory responsivity in autism. While contrast reliably modulated responses across all experiments, group differences were observed specifically in SSVEP amplitudes. Autistic individuals exhibited heightened SSVEP responses in comparison to their non‐autistic counterparts, yet these differences were not accompanied by statistically significant group differences in hemodynamic or perceptual responses in the present sample. These findings highlight SSVEPs as a sensitive index of group differences in temporal processing of visual stimuli. The relevance of SSVEP amplitude was further supported by its association with low‐contrast BOLD responses and self‐reported hypersensitivity, linking frequency‐locked neural responses to both visual cortical activity and subjective sensory experience. Future research should aim to further characterize the temporal dynamics of sensory processing in autism and relate those to autistic individuals' sensory experiences.

## Funding

This work was supported by National Institutes of Health (R01MH118847) awarded to S.O.M. and S.J.W., NEI Vision Training Grant 5T32EY007031 awarded to D.L.S., Simons Foundation, SFARI grant 647415 awarded to S.O.M. and S.J.W.

## Ethics Statement

All study protocols were approved by the Institutional Review Board of the University of Washington.

## Consent

All participants provided written informed consent prior to participation.

## Conflicts of Interest

The authors declare no conflicts of interest.

## Supporting information


**Supplemental Table 1** Additional demographic characteristics of sample. Further demographic information regarding race and ethnicity, and endorsed mental health concerns per group.
**Supplemental Figure 1**. Effect of psychotropic medication. SSVEP amplitudes (±SEM) in autistic participants as a function of SSRI or stimulant use, and contrast condition. No significant main effects of medication use or medication‐by‐contrast interactions were observed.
**Supplemental Figure 2**. Associations between SSVEP amplitude and sensory sensitivity scores considering ADHD diagnosis. Scatterplot showing the correlation between SSVEP amplitude and hypersensitivity scores in non‐autistic and autistic adults. Individual data points are further differentiated by ADHD diagnosis. Notably, ADHD status is represented across all quadrants.

## Data Availability

Data is available through the NIMH Data Archive (NDA) at https://doi.org/10.15154/qzkh‐3v06.
