# Supplementary material for: Visual Responsivity in Autism: Measuring Visual Responses in Autistic and Non‐Autistic Adults Using Psychophysics, fMRI, and EEG
Source: Autism Res. 2026 Jul 31;19(9):e70335. doi: 10.1002/aur.70335 (PMC13599697; doi:10.1002/aur.70335)
Supplement: Supplementary file 1 [file AUR-19-0-s001.docx]

*Supplemental Analysis 1. Validation of Low Contrast Neural Responses*

To confirm that the low contrast (2%) flickering stimulus elicited measurable neural responses rather than reflecting an absence of stimulus presentation, we conducted additional baseline comparisons for both EEG and fMRI measures.

For the EEG steady-state visual evoked potential (SSVEP) response, SSVEP amplitude at the fundamental frequency was defined as the maximum amplitude within 5.5–6.5 Hz. Baseline spectral noise was estimated as the mean amplitude of neighboring frequency bins (5–5.5 Hz and 6.5–7 Hz). Baseline-corrected SSVEP amplitudes were computed by subtracting the noise estimate from the 6 Hz amplitude for each participant. One-sample t-tests against zero demonstrated that SSVEP amplitudes in the low contrast condition were significantly greater than baseline spectral noise (t(51) = 8.17, p < .0001, Cohen’s d = 1.13), indicating reliable stimulus-locked neural entrainment despite reduced response magnitude.

For the fMRI BOLD responses, percent signal change was averaged across stimulus blocks and ROIs (V1–V3) for each participant in the low contrast condition. One-sample t-tests against zero showed that low contrast stimuli elicited BOLD responses significantly greater than baseline (mean percent signal change = 1.37%; t(56) = 22.87, p < .0001, Cohen’s d = 3.03), confirming the presence of measurable hemodynamic activation. Regions of interest were defined using high contrast localizer activation, and percent signal change for both contrast conditions was extracted from these independently defined ROIs, avoiding circularity in tests of low contrast responses.

Together, these analyses demonstrate that the low contrast condition elicited reliable neural responses across modalities, supporting its interpretation as a weak but detectable stimulus rather than an absence of visual input.

*Supplemental Analysis 2. Sensitivity Analysis of Neural Responses*

To assess relative measurement sensitivity across EEG and fMRI methods, we evaluated split-half reliability for SSVEP and BOLD responses. For SSVEP, we split trials into odd and even halves, computed 6Hz amplitude for each half separately, and correlated these halves across participants. SSVEP amplitudes at the fundamental frequency showed excellent split-half reliability in both high and low contrast conditions (r=.93 in both conditions). Using an analogous approach for fMRI, we computed block-split reliability by averaging independent sets of stimulus blocks across runs, collapsing responses across early visual ROIs, and correlating the resulting split-half estimates across participants. For fMRI, block-split reliability revealed high reliability in the high contrast condition (r=.86) and moderate-to-high reliability in the low contrast condition (r=.69). These results indicate that while both modalities yielded reliable responses, SSVEP measures exhibited consistently higher reliability, particularly at low contrast.

*Supplemental Analysis 3. Power Analyses*

We computed sensitivity estimates separately for each method based on sample size variation across experiments. Using two-sample power calculations (α = .05, two-sided; 80% power), the minimum detectable between-group effect sizes were d = .77 for psychophysics (n=30 autistic, n=25 non-autistic), d = .76 for fMRI (n=30 autistic, n=27 non-autistic), and d = .80 for EEG (n=29 autistic, n=23 non-autistic). These estimates indicate that the study was powered to detect medium-to-large between-group effects, but was underpowered to detect small group differences. Accordingly, null group effects should be interpreted cautiously as they do not rule out subtle differences. Notably, the observed SSVEP group effect (d=.27) falls below the minimum detectable effect size estimated for EEG (d = .80), underscoring that this estimate may be imprecise despite statistical significance and should be interpreted with appropriate caution.

| **Measure** | **Autistic**  N (%) | **Non-autistic**  N (%) |
| --- | --- | --- |
| **Race and ethnicity** |  |  |
| Asian | 0 (0%) | 9 (33.3%) |
| Black/African American | 0 (0%) | 1 (3.7%) |
| White | 26 (83.9%) | 14 (51.9%) |
| Biracial | 5 (16.1%) | 3(11.1%) |
| Hispanic/Latino | 5 (16.1%) | 1 (3.7%) |
|  |  |  |
| **Endorsed mental health concerns** |  |  |
| Anxiety | 22 (71.0%) | 1 (3.7%) |
| Depression | 17 (54.8%) | 4 (14.8%) |
| OCD | 5 (16.1%) |  |
| Eating disorders | 5 (16.1%) |  |
| PTSD | 3 (9.7%) |  |
| Substance use | 2 (6.5%) |  |
| Other | 1 (3.2%) |  |

*Supplemental Table 1. Additional demographic characteristics of sample.* Further demographic information regarding race and ethnicity, and endorsed mental health concerns per group.

*Supplemental Figure 1. Effect of psychotropic medication.* SSVEP amplitudes (±SEM) in autistic participants as a function of SSRI or stimulant use, and contrast condition. No significant main effects of medication use or medication-by-contrast interactions were observed.

*Supplemental Figure 2. Associations between SSVEP amplitude and sensory sensitivity scores considering ADHD diagnosis.* Scatterplot showing the correlation between SSVEP amplitude and hypersensitivity scores in non-autistic and autistic adults. Individual data points are further differentiated by ADHD diagnosis. Notably, ADHD status is represented across all quadrants.
